# Genetic Architecture of Untargeted Lipidomics in Cardiometabolic-Disease Patients Combines Strong Polygenic Control and Pleiotropy

**DOI:** 10.3390/metabo12070596

**Published:** 2022-06-27

**Authors:** Francois Brial, Lyamine Hedjazi, Kazuhiro Sonomura, Cynthia Al Hageh, Pierre Zalloua, Fumihiko Matsuda, Dominique Gauguier

**Affiliations:** 1 Center for Genomic Medicine, Graduate School of Medicine Kyoto University, Kyoto 606-8501, Japan; francois.brial@free.fr (F.B.); fumi@genome.med.kyoto-u.ac.jp (F.M.); 2 INSERM UMR 1124, Université Paris Cité, 45 rue des Saint-Pères, 75006 Paris, France; 3 Beemetrix SAS, 30 Avenue Carnot, 91300 Massy, France; lhedjazi@beemetrix.com; 4 Life Science Research Center, Technology Research Laboratory, Shimadzu Corporation, Kyoto 606-8501, Japan; kazuhiro.sonomura@genome.med.kyoto-u.ac.jp; 5 College of Medicine and Health Sciences, Khalifa University, Abu Dhabi P.O. Box 17666, United Arab Emirates; cynthia.alhageh@ku.ac.ae (C.A.H.); pierre.zalloua@ku.ac.ae (P.Z.); 6 McGill University and Genome Quebec Innovation Centre, 740 Doctor Penfield Avenue, Montreal, QC H3A 0G1, Canada

**Keywords:** lipidomics, coronary artery disease, genetics, metabotypes, molecular phenotyping, GWAS, MWAS, SNP

## Abstract

Analysis of the genetic control of small metabolites provides powerful information on the regulation of the endpoints of genome expression. We carried out untargeted liquid chromatography–high-resolution mass spectrometry in 273 individuals characterized for pathophysiological elements of the cardiometabolic syndrome. We quantified 3013 serum lipidomic features, which we used in both genome-wide association studies (GWAS), using a panel of over 2.5 M imputed single-nucleotide polymorphisms (SNPs), and metabolome-wide association studies (MWAS) with phenotypes. Genetic analyses showed that 926 SNPs at 551 genetic loci significantly (q-value < 10^−8^) regulate the abundance of 74 lipidomic features in the group, with evidence of monogenic control for only 22 of these. In addition to this strong polygenic control of serum lipids, our results underscore instances of pleiotropy, when a single genetic locus controls the abundance of several distinct lipid features. Using the LIPID MAPS database, we assigned putative lipids, predominantly fatty acyls and sterol lipids, to 77% of the lipidome signals mapped to the genome. We identified significant correlations between lipids and clinical and biochemical phenotypes. These results demonstrate the power of untargeted lipidomic profiling for high-density quantitative molecular phenotyping in human-genetic studies and illustrate the complex genetic control of lipid metabolism.

## 1. Introduction

Molecular-phenotyping tools based on transcriptome, proteome and metabolome technologies provide detailed information on the molecular pathways and biomarkers relevant to disease etiopathogenesis. Their application in the context of genome-wide association studies (GWAS) of complex disorders can enhance our understanding of the genetic control of genome expression and to dissect out disease variables into multiple, intermediate disease traits and molecular phenotypes [1,2]. Metabolomics, which analyses the multivariate data representing a range of small metabolites in a biological sample, has already been used in humans to map the genetic determinants of the quantitative variations of metabolites [3]. Owing to the role of altered plasma-lipid profiles in many chronic-disease manifestations, including chronic kidney disease, cardiovascular risk, dyslipidemia and neurological disorders, the detection and quantification of lipids in a biospecimen through lipidomics has emerged as a promising approach to correlate variations in blood lipids with these diseases [4,5,6].

Even though elevated blood LDL cholesterol is known to be a major risk factor for coronary heart disease and stroke, lipidomics enables a hypothesis-free strategy for broadening the search for the biomarkers associated with these diseases to a wide range of lipid species and to uncover novel targets beyond traditional lipids that can predict or reduce the risk of cardiovascular diseases [7,8]. Among examples of lipid classes that can be detected and quantified through lipidomic technologies, ceramides are involved in vascular inflammation and apoptosis and may have a higher potential to predict coronary heart disease than LDL cholesterol [9]. Ceramides, but more prominently the phospholipid species, alter the progression to ischemic cardiomyopathy [10]). Beyond associations between lipids and disease, combining genetics and lipidomics allows the identification of the genetic factors involved in the coordinated regulation of lipid species, thus inferring functional connections between different lipid species and causal relationships between lipid species and disease status or disease endophenotypes. The most robust GWAS studies of blood-lipid metabolism have focused on circulating total, LDL and HDL cholesterol and triglycerides, which are easily quantified using standard, clinical chemistry assays [11,12]. The extension of GWAS to deeper analyses of lipid species requires mass-spectrometry (MS) technologies and analytical methods that allow for the enhanced efficiency and coverage of lipidome profiling [13]. The application of MS-based lipidomics to GWAS was initially based on targeted analysis of blood sphingomyelins and ceramides [14] and was recently extended to increasing numbers of known lipids [15,16].

Here, we applied liquid chromatography–mass spectrometry (LC–MS) to a group of 273 individuals well-characterized for clinical and biochemical phenotypes relevant to cardiometabolic diseases, to analyse the genetic architecture of lipid metabolism in humans. We were able to identify evidence of the pleiotropy and strong polygenic control of lipids and proposed annotations for lipidomic signals mapped to the human genome. This study demonstrates the power of untargeted lipidomics for high-density quantitative molecular phenotyping in humans and illustrates the complex genetic control of blood-lipid metabolism.

## 2. Results

### 2.1. Clinical-Data Analysis

The study group has a mean age of 57.4 ± 0.7 years and 56.4% (*n* = 154) of the individuals were males (Table 1). All individuals in the cohort were devoid of evidence of coronary artery stenosis, as assessed by an angiogram analysis. Analyses of the pathophysiological components of the cardiometabolic syndrome revealed that 132 individuals (49%) were obese (BMI > 30 kg/m^2^), 46 had type 2 diabetes (17%), 147 were hypertensive (54%) and 119 were hyperlipidemic (44%), with a similar proportion of affected males and females (Table 2).

### 2.2. General Features of Untargeted-Lipidome Data

Untargeted-lipidome profiling retrieved 3013 spectral features characterized by a specific mass-to-charge ratio (*m*/*z*) and retention time (RT) (1529 in the negative-ionization mode and 1484 in the positive-ionization mode) that met the acceptance criterion (i.e., Relative Standard Deviation (RSD) < 30%, also referred to as Coefficient of Variation CV) (Supplementary Table S1). Multivariate Principal Component Analysis (PCA) analysis showed the absence of strong technical drift during spectral-data acquisition in the cohort, as illustrated by the PCA scores’ 2D plot representation of the QC samples in the two ionization modes (Supplementary Figure S1). The QC samples were tightly clustered, which indicates an acceptable reproducibility of the retained set of metabolic features as well as a good stability of the LC–MS-profiling experiments.

### 2.3. General Features of Untargeted-Lipidome Data

**Figure 1:**
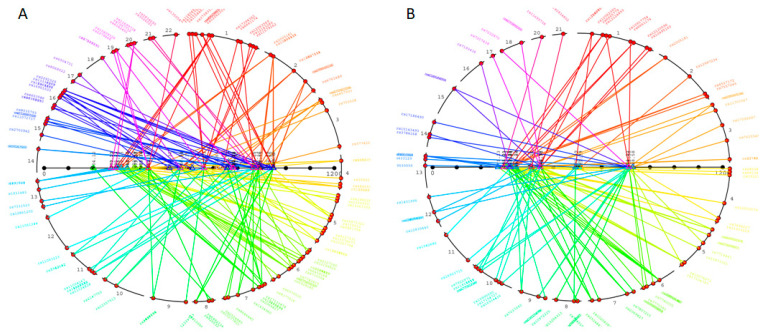
Genome-wide association study of metabolomic features (mGWAS) in the study group. Data are shown for metabolic features acquired in positive (**A**) and negative (**B**) ionization modes, showing evidence of significant association (LOD > 8) with an SNP locus. Chromosomes are color-coded on the circle. The colors of the lines indicate the chromosomal location of SNP loci showing evidence of significant association with metabolic features, characterized by a mass-to-charge ratio (horizontal axes). Details of genetic results are given in Supplementary Table S2.

Genome-wide association of untargeted-lipidome-profiling data identified 5501 statistically significant associations (FDR-adjusted p-value; q-value < 10^−8^) between SNPs and spectral features (1905 in the negative ionization mode and 3596 in the positive ionization mode). Further analyses of lipid features and their isotopes reduced the analyses to 926 significant associations, between 551 distinct SNP loci and apparently independent lipidome features (Figure 1) (Supplementary Table S2). Eventually, only 74 lipidome features showed evidence of statistical association (q-value < 10^−8^) to a genetic locus in the cohort (25 in the negative ionization mode and 49 in the positive ionization mode) (Table 3).

**Figure 2:**
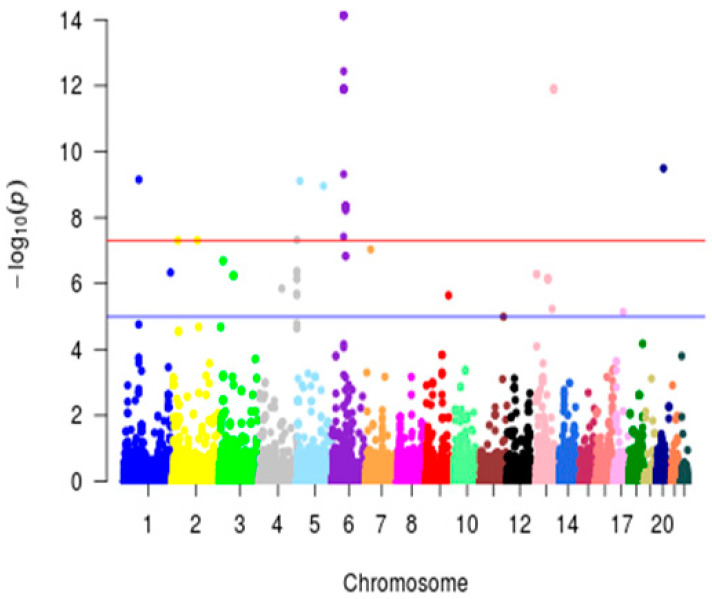
Manhattan plot illustrating the polygenic control of metabolic features. Genome-wide association study was carried out with over 2.5 M imputed SNPs, for the metabolomic feature characterized by a mass-to-charge ratio of 227.216 and a retention time of 67.49. Chromosomes are color-coded. Evidence of significant associations (LOD >8) with this metabolic feature were found on chromosomes 1, 5, 6, 13 and 20. The *Y*-axis corresponds to the significance of the association (−Log10 *p*-values). The *X*-axis represents the physical location of the variant colored by chromosome.

**Figure 3:**
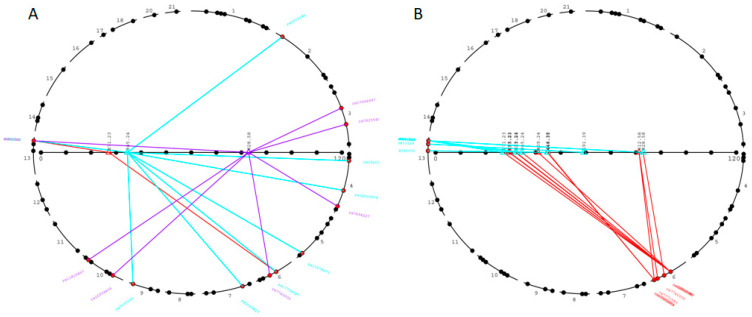
Architectural characteristics of genetic associations to metabolic features. Evidence of polygenic control of metabolites (**A**) and potential pleiotropic effects of genetic loci on metabolite abundance (**B**) were identified, following metabolomic analysis of serum samples of 273 individuals. The colours of the lines indicate the chromosomal location of SNP loci showing evidence of significant association (LOD > 8), with the abundance of a specific metabolic feature. Evidence of polygenic control of the abundance of metabolic features was found for compounds characterized by mass-to-charge ratios (horizontal axis) of 271.23 (red), 345.24 (blue) and 828.58 (purple) (**A**). Potential pleiotropic effects were detected for SNP loci on chromosomes 6 (red lines) and 13 (blue lines), significantly associated with metabolic features characterized by distinct mass-to-charge ratios on the horizontal axis (**B**). Details of genetic results are given in Supplementary Table S2.

Evidence of polygenic control was observed for 52 lipidome features (Table 3), as illustrated with the compound detected, *m*/*z*: 277.22 (negative-ionization mode), which was controlled by genetic loci in chromosomes 6 (rs7749100 in DST, q-value = 1.903 × 10^−13^), 13 (rs1410818, q-value = 4.31 × 10^−10^) and 20 (rs11699738 in SOGA1, q-value = 4.75 × 10^−9^) (Figure 2, Supplementary Table S2). Such strong polygenic regulations of lipid metabolism are further illustrated in Figure 3A, with the associations of *m*/*z* 271.23, 345.24 and 828.58 (negative-ionization mode), with multiple distinct genetic loci. The compound characterized by an *m*/*z* of 345.24 was significantly associated with eight distinct genetic loci on chromosomes 2 (rs2005181 in BABAM2, q-value = 5.68 × 10^−10^), 4 (rs292037, q-value = 1.93 × 10^−13^ and rs12500579 in ANK2, q-value = 4.24 × 10^−9^), 5 (rs10076673 in PITX1, q-value = 7.40 × 10^−12^), 6 (rs7749100 in DST, q-value = 3.11 × 10^−10^), 7 (rs2069827 in STEAP1B, q-value = 1.23 × 10^−11^), 9 (rs7037093, q-value = 2.59 × 10^−12^) and 13 (rs1410818, q-value = 1.38 × 10^−14^) (Figure 3A, Supplementary Table S2).

The remaining 22 lipidomic features exhibited evidence of monogenic control. For example, several lipidomic signals acquired by the positive-ionization mode were controlled by a single marker locus on chromosomes 2 (rs12997234 in DPP10 with *m*/*z* 568.340 and 590.3213), 3 (rs2002218 in IQSEC1 with *m*/*z* 712.645), 5 (rs13362253 in MSX2 with *m*/*z* 766.574), 6 (rs7759479 in DST with *m*/*z* 279.232, rs6928180 in GRIK2 with *m*/*z* 344.279, 370.295, 398.326, 400.342 and 426.357, rs1009439 in RCAN2 with *m*/*z* 377.266 and *m*/*z* 379.282), 8 (rs6992234 with *m*/*z* 204.123), 15 (rs11855528 in CMIP with *m*/*z* 612.556 and rs11071737 in RAB8B with *m*/*z* 932.864), 16 (rs2292329 in NECAB2 with *m*/*z* 922.785) and 20 (rs2260930 in SEL1L2 with *m*/*z* 780.553) (Table 3).

### 2.4. Genetic Analysis of Lipid Metabolism Uncovers Evidence of Pleiotropy

We identified 44 SNP loci that control two or more metabolic features, indicating potential pleiotropic effects of genetic variants, as illustrated in Figure 3B, where closely linked SNPs on chromosomes 6 and 13 are associated with a different *m*/*z*. For example, the above-mentioned SNP rs6928180 in GRIK2 was associated with several lipidome features under monogenic control (*m*/*z* 344.279, q-value = 1.89 × 10^−23^; *m*/*z* 370.295, q-value = 1.14 × 10^−32^; *m*/*z* 398.326, q-value = 4.96 × 10^−34^; *m*/*z* 400.342, q-value = 3.68 × 10^−28^; *m*/*z* 426.357, q-value = 7.38 × 10^−18^) suggesting a pleiotropic effect of variants in GRIK2 on distinct but coordinately regulated lipids (Table 3). Along the same line, marker rs12997234 on chromosome 2 in an intron of DPP10 was exclusively associated with the monogenic control of *m*/*z* 568.34 (q-value = 1.73 × 10^−11^) and *m*/*z* 590.32 (q-value = 2.93 × 10^−17^) in the positive-ionization mode and with *m*/*z* 612.33 (q-value = 1.46 × 10^−9^) in the negative-ionization mode (Table 3). The most striking example of pleiotropy was detected on chromosome 13 at the locus rs1410818 and 11 distinct *m*/*z* values (Supplementary Table S2).

### 2.5. Assignment of Lipids to Lipidomic Features Mapped to the Human Genome

We next carried out the identification of candidate lipids for each of the 74 features showing evidence of genetic control. Using the LIPID MAPS database, we were able to annotate 26 lipidome signals with a single lipid, including 10 which were controlled by a single genetic locus (Table 3). Several lipid candidates could be proposed for the remaining 48 lipidome features, which prevented the unambiguous assignment of lipids. The vast majority of assigned lipids were fatty acyls (27), sterol lipids (23), triacylgycerols (9) and, to a lesser extent, a combination of phosphatidylcholines, phosphatidylethanolamine and phosphatidylserines (20).

### 2.6. Metabolome-Wide Association Studies Identify Metabolites Associated with Clinical and Biochemical Phenotypes

**Figure 4:**
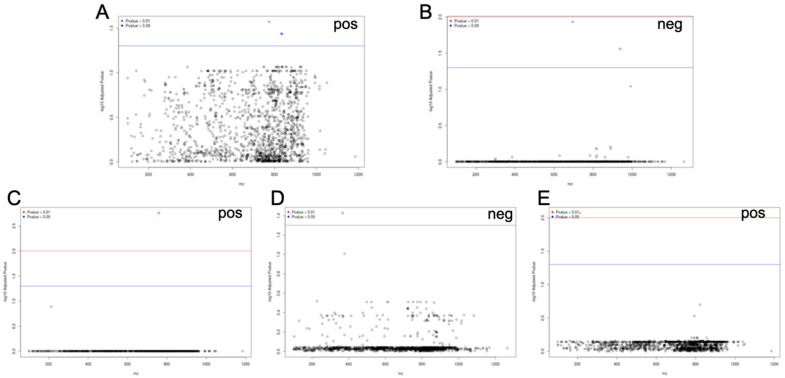
Metabolome-wide association studies (MWAS) in patients with cardiometabolic syndrome. Correlations were tested between clinical and biochemical phenotypes and serum metabolic features characterized by a mass-to-charge ratio (*m*/*z*) shown on the x-axes. Data are shown for body-mass index (**A**), family history of hypertension (**B**), total cholesterol (**C**) and HDL cholesterol (**D**,**E**). The *Y*-axis corresponds to the adjusted false-discovery rate (FDR). Regression analysis was adjusted for age and sex effects by including them as covariates in the model. pos, positive ionization mode; neg, negative ionization mode.

To test for evidence of association between clinical and variations in biochemical phenotypes and compounds from the lipidome dataset mapped to the human genome, linear regression was performed. Results from associations with a nominal *p* < 0.05 are given in Supplementary Table 3. Significant associations (q-value < 0.05) with multiple metabolic features were detected for cardiometabolic disease (Table 4). Fewer significant associations were identified for family history of hypertension (*m*/*z* 695.511 and 938.536) and for variations in body-mass index (*m*/*z* 774.543, 833.588, 834.591 and 832.584), total cholesterol (*m*/*z* 758.569 and 759.572) and HDL cholesterol (*m*/*z* 367.228 and 213.146) (Figure 4, Table 4). Family history of diabetes also showed evidence of marginal association to the feature *m*/*z* 695.511 (nominal *p*-value = 0.036) (Supplementary Table S3). Associations to family history of hypertension and diabetes independent to association to the diseases suggest that the underlying lipidomic feature may be a predictive marker of both diseases.

We did not identify statistically significant associations to LDL cholesterol or triacylglycerols. However, over 60 lipidomic features showed marginal evidence of co-association (nominal *p*-value < 0.05) to both LDL and HDL cholesterol (e.g., *m*/*z* 129.98 and 171.99) and five features (*m*/*z* 213.15, 367.23, 367.26, 369.27 and 722.50) were marginally associated to triacylglycerols and total, HDL and LDL cholesterol (Supplementary Table S3). No significant associations were found between spectral data and other phenotypes. We were able to assign one or several putative lipids to 14 lipidome signals, including ST 27:2;O;Hex and ST 28:1;O5, which were found to be regulated by multiple genetic loci (Table 4).

## 3. Discussion

We report results from the genome mapping of untargeted serum lipidomics in a group of individuals characterized for pathophysiological features of the cardiometabolic syndrome. We identified evidence of strong polygenic control of lipid features and instances of mechanisms of pleiotropy in the regulation of lipid metabolism. These observations illustrate the complex genetic architecture of serum lipid regulation and provide novel information beyond the genetic control of cholesterol metabolism.

Both proton nuclear magnetic resonance (^1^H NMR) and mass spectrometry (MS) have been successfully used to map the genetic control of predominantly serum metabolites in genome-wide association studies (GWAS) in humans [17]. Collectively, over 1800 metabolomic data (i.e., known and unknown metabolites and ratios) have been associated with over 40,000 unique SNPs [18]. Among these, MS-lipidomic data provide significant advances in our understanding of the etiopathogenesis of diseases characterized by anomalies in lipid metabolism [19]. Untargeted lipidomics, a hypothesis-free strategy that has the power of deepening quantitative lipid analyses to unassigned lipids, remains challenging due to the breadth and intrinsic complexity of known lipids, which differ in terms of physicochemical properties [13,20]. As a consequence, harmonization of sample preparation for such a heterogeneous group of molecules is a problematic issue that limits detection and quantification of the broad diversity of lipid species [21]. In addition, variations in MS-instrument stability affect repeatability within and between experiments. Finally, the unambiguous assignment of putative lipids to MS-spectral signals remains an important methodological consideration in the application of untargeted MS lipidomics in GWAS.

Polygenic control is a hallmark of GWAS of human chronic diseases and complex phenotypes, and the genetic regulation of metabolomic profiling data does not make any exceptions [22,23,24]. We show that serum-lipid abundance exhibits predominant polygenic control, when a single metabolite is associated with several unlinked SNPs. Results from lipidomic GWAS have shown that about 30% of lipids are associated with several genetic loci [16]. Specifically, loci on chromosomes 2 and 4 control triglyceride TAG(50:1;0), loci on chromosomes 8 and 11 are associated with triglyceride TAG(52:3;0) and loci on chromosomes 12 and 18 control lysophosphatidylcholine LPC(14:0;0) [15]. This pattern of polygenic control suggests either functional redundancy of proteins in the regulation of lipid metabolic pathways, or the involvement of distinct proteins each contributing in parallel or in concert to interconnected mechanisms of lipid sensing, synthesis, transport and degradation.

Our association results also suggest apparent pleiotropy when a single genetic locus controls multiple, different lipidomic features. It is expected to occur in metabolic processes, since altered regulation of an individual protein involved in an enzymatic reaction or metabolite binding or transport may result in changes in interconnected biological pathways affecting multiple metabolites. An excess of distinct lipid species associated with genomic regions in lipidomic GWAS suggests the widespread occurrence of this phenomenon in the regulation of lipid metabolism [15,23,24]. Harshfield et al. reported the genetic mapping of 181 lipids to only 24 genomic regions [16], and Tabassum et al. identified associations to 42 lipid species in 11 genomic regions [15], thus implying that one genomic region is associated with several lipids. One of the most striking examples of pleiotropy in lipidomic GWAS is the GCKR locus, which is associated with over 30 lipid species [25]. The eicosanoid metabolic network, which involves 28 proteins for the production of over 150 lipids, provides a further example of pleiotropy in the regulation of lipid biology [26]. These coordinately regulated lipid clusters suggest the existence of genetically-determined “lipidotypes”.

Combined with clinical data, lipidomic-based phenotyping allows the definition of disease-associated biomarkers as well as druggable-metabolite targets. Integrating genotyping data can identify instances of co-localization of disease-risk SNPs and loci associated with metabolomic features, which may represent disease-causative molecular biomarkers [15,16,27]. With the exception of SEL1L2 and SYT9, gene loci showing evidence of monogenic control of lipids in our study have been associated with disease-relevant phenotypes (e.g., body mass index), biochemical variables (e.g., creatinine) and behavioral traits in the GWAS repository (www.ebi.ac.uk/gwas/, accessed on 1 May 2022). Interestingly, multiple SNPs, the locus of the gene encoding pleckstrin and the Sec7 domain containing 3 (PSD3), which controls the level of a carnitine in our study, have been consistently associated with triglycerides and cholesterol levels as well as type 2 diabetes and obesity [28], and their downregulation results in reduced hepatic lipids in vitro and protects against fatty liver in vivo in mice [29].

Considering the breadth of circulating lipid species [7,21] and their roles in cardiovascular diseases [19], we were able to map the genetic control of several lipid species, mostly fatty acyls, phospholipids and triglycerides. On the other hand, we were unable to identify genetic loci associated with several important lipid species, including, for example, sphingomyelins and ceramides, which are involved in cardiovascular risk [9,30]. This may be caused by technical issues with data acquisition and the relatively modest sample size of the study but may also be accounted for by specific clinical features of the individuals selected in our study. Absence of coronary-artery stenosis in these individuals suggests reduced cardiovascular risk and, therefore, potentially limited quantitative variations in blood ceramides in cases and controls that may prevent genetic mapping. In support of this hypothesis, we did not identify statistically significant associations between lipidomic features and hypertension, which might nevertheless be improved with the use of intermediate, quantitative phenotypes, including measures of blood pressure. In addition, the fact that CMD patients may be under various medications, including lipid-lowering drugs (statins) or anti-diabetic treatments that result in improved control of blood pressure [31], may explain the absence of statistically significant associations between lipidomic features and hypertension in our study. However, our results suggest a role of lipids in the family history of hypertension, which may represent disease-predictive markers.

## 4. Materials and Methods

### 4.1. Study Subjects

The study group consisted of 273 subjects selected from a larger study recruited between 2006 and 2009 for inclusion in the FGENTCARD patient collection, primarily designed to map the genetic determinants of coronary artery stenosis [32]. Individuals from the FGENTCARD cohort were originally referred to a catheterization care unit for clinical evaluation. A 20 mL blood sample was collected in overnight fasted individuals from the peripheral femoral artery during the coronary angiography for serum preparation. Patients provided a written consent for the whole study including genomic analyses. The Institutional Review Board (IRB) at the Lebanese American University approved the study protocol.

Body weight, body-mass index (BMI) and blood chemistry (total, HDL and LDL cholesterol, triglycerides) were determined. Evidence of diabetes (fasting glucose > 125 mg/dl), hypertension (blood pressure > 10/14 mm Hg) and obesity (BMI > 30) was recorded in individuals’ medical charts. Evidence of cardiometabolic disease (CMD) was assessed by presence of at least three anomalies (diabetes, hypertension, BMI > 30 kg/m^2^ and HDL < 40 mg/dl). All 273 individuals selected for this genetic study were devoid of vessel stenosis, assessed through coronary angiography carried out at a single recruitment site. Family history of diabetes and hypertension, defined by presence of the disease in a sibling, parent or second-degree relative, was also recorded.

Statistical analysis of clinical and biochemical data was performed using two-way ANOVA. Differences were considered statistically significant with a *p* < 0.05.

### 4.2. Chemicals

Isopropanol, acetonitrile, formic acid and ammonium formate were LC–MS Chromasolv^®^ Fluka and high-performance liquid chromatography (HPLC) quality and were purchased from Sigma-Aldrich (Sigma-Aldrich, Saint-Quentin Fallavier, France). Ultra-pure water (resistivity: 18 mΩ) was obtained with a Milli-Q Integral purification system (Millipore, Molsheim, France) fitted with a 0.22 µm filter. The mobile phase was prepared with a solvent containing 400 mL of water, 600 mL of acetonitrile, 0.1% formic acid and 0.630 g of ammonium formate, and a solvent containing 100 mL of acetonitrile, 900 mL of isopropanol, 0.1% formic acid and 0.630 g of ammonium formate.

### 4.3. Sample Preparation

Lipid extraction from serum was performed using isopropanol (1:6, *v/v*), as recommended by the MS-equipment supplier, which is the most robust solvent enabling a broad coverage and recovery of lipid species from serum [33]. Experiments were carried out with 50 µL serum aliquots. Samples were then centrifuged at 14,000 g, and supernatants were then transferred to vials for injection in the UPLC system.

### 4.4. UPLC analysis

A Waters Acquity UPLC^®^ (Waters Corp, Saint-Quentin en Yvelines, France) fitted with a Acquity CSH C18 column (2.1 × 150 mm, 1.7 µm) and a corresponding guard column (Acquity CSH 1.7μM) (Waters Corp, Saint-Quentin en Yvelines, France) were used to analyse lipid compounds in serum samples as previously described [34]. The oven temperature was set at 55 °C. The flow rate used for these experiments was 400 µL/min and a volume of 5 µL of sample was injected. The total run time was 24 min. A binary gradient consisted of above-described mobile phases was used according to Waters’ recommendation. Mobile phase B was maintained at 99% during 4 min at the end of the gradient.

### 4.5. Mass Spectrometry

Mass spectrometry was carried out as previously [34]. The UPLC system was coupled with a Q-Exactive™ Hybrid Quadrupole-Orbitrap mass spectrometer (Thermo Fisher Scientific, Illkirch, France). Infusion of a calibration mixture (caffeine, MRFA and Ultramark^®^ 1621) was used for calibration of the instrument. Parameters of the heated-electrospray (HESI-II, Thermo Fisher Scientific, Illkirch, France) interface were as follows: S-Lens 50 V, Sheat gas: 65, Auxiliary gas: 25 arbitrary units, capillary voltage 3 kV, capillary temperature 350 °C and vaporization temperature 60 °C. The maximum target capacity of the C-trap (autogain control, AGC) target was defined as 3e6 ions and the maximum injection time was 200 ms. Full scans were obtained in positive and negative ion modes simultaneously with a resolution of 70,000 full width at half maximum (FWHM), in the scan range of mass-to-charge ratio (*m*/*z*) of 85–1275.

### 4.6. Untargeted Lipidomic Data Analysis

Analysis of MS data derived from UPLC complied with standard protocols and food and drug administration (FDA) guidelines [35,36], as previously described (34). XCMS tools implemented in R statistical language (v 3.1.0) (http://www.bioconductor.org, accessed on 10 May 2020) were used for preprocessing steps of MS data analysis (peak picking, peak grouping, retention-time correction, annotation of isotopes and adducts). Profiles of positive and negative ionization modes were separately extracted and converted into mzXML format for preprocessing by the XCMS tools. Identification of Regions of Interest (ROI) used the wavelet-based peak-picking approach (centwave). MS-data preprocessing resulted in a peak table listing lipidomic features characterized by a retention time (RT), mass-to-charge ratio (*m*/*z*) and corresponding intensity for each serum sample.

A data matrix reduction was applied to retain spectral features consistently found in the individuals. Over 40% of missing values were withdrawn. Performance and reliability of the analytical process and compliance of data with FDA-acceptance criteria [37] were also verified through a quality assurance (QA) strategy, based on analysis of a pooled quality-control (QC) sample, which was injected every 10 samples throughout the analytical run. Median fold-change-normalization approach [38] was applied on the retained MS features, followed by a generalized log-transformation. A threshold of 30% calculated for each metabolic feature in the QC samples was set for relative standard deviation (RSD), which is an accepted standard to assess data reproducibility in metabolomic studies [35,36]. Four samples were identified as outliers and were discarded from the study. The resulting matrix was then used for multivariate and univariate statistical analyses (principal component analysis and linear regression).

### 4.7. Metabolome-Genome Wide Association Studies (mGWAS)

All individuals were genotyped by Illumina Human610-Quad BeadChip and Illumina Human660W-Quad BeadChip, respectively (552,510 overlapping SNPs), as part of the FGENTCARD consortium [32]. All SNPs with over 98% genotyping success rate, minor allele frequency above 1% and in Hardy-Weinberg equilibrium (*p*-value > 1 × 10^−7^) were included in the analysis. An imputation across the whole genome to CEU HapMap population as a reference was performed using the IMPUTE2 tool [39], which yielded 2,573,690 SNPs. The plink tool [40] was used to perform both association analyses based on an additive genetic model. An FDR adjusted *p*-value (q-value) < 1 × 10^−8^ was considered to be significant genome-wide. Plotting circles were generated using an in-house tool specifically developed to illustrate mGWAS associations.

### 4.8. Metabolome-Wide Association Studies (MWAS)

A linear-regression model was applied to carry out MWAS through the assessment of association, between each metabolic feature with clinical and biochemical continuous phenotypes (total, HDL and LDL cholesterol, triglycerides). Normality assumption of the residuals of each metabolic feature was investigated by Shapiro–Wilk test. The R statistical language was used to perform the linear regression and compute a *p*-value for each metabolic feature with a threshold of significance set to 0.05. Adjustment for age and sex was performed by including them as covariates in the statistical model. False discovery rates (FDR) were corrected using the Benjamini-Hochberg method to adjust P-values for false discovery involving multiple comparisons.

### 4.9. Assignment of Lipid Features

Annotation of lipid candidates corresponding to lipidome signals was carried out using the free resource LIPID MAPS (https://www.lipidmaps.org, accessed on 1 May 2022). We initially performed bulk-structure searches and subsequently refined our analysis by interrogating the LIPID MAPS Structure Database (LMSD) with a list of precursor ions. We entered the list of precursor ion *m*/*z* and chose appropriate polarity for the adduct ions. We defined a mass tolerance of ±0.001 *m*/*z* and sorted our data according to the delta between the input *m*/*z* and the *m*/*z* of candidate proposed in the database.

## 5. Conclusions

Results from our untargeted-lipidomic profiling provide information on fundamental mechanisms regulating serum lipids in humans. Replication of these findings in larger study populations and further analyses, such as MS/MS validation experiments designed to unambiguously assign lipids to lipidomic features, are required.

## Figures and Tables

**Table 1:** Clinical and biochemical features of individuals in the study group used for metabolomic profiling. Individuals were selected for absence of coronary stenosis. Data are given as means ± SEM. Number of cases are reported in parentheses. Gender differences were tested using two-way ANOVA.

	All		Females		Males	
	Mean	Range	Mean	Range	Mean	Range
Age	57.4 ± 0.7 (273)	30–83	61.4 ± 0.9 (119)	38–83	54.4 ± 0.9 (154)	30–81
Body weight (kg)	83.13 ± 0.99 (269)	50–150	77.69 ± 1.44 (118)	52–150	87.39 ± 1.26 (151)	50–130
BMI (kg/m^2^)	30.37 ± 0.33 (268)	18.96–55.77	31.36 ± 0.56 (118)	20.34–55.77	29.59 ± 0.37 (150)	18.96–44.29
Glucose (mg/dL)	107.95 ± 2.19 (219)	60–299	111.41 ± 3.98 (98)	62–299	105.14 ± 2.29 (121)	60–255
Total cholesterol (mg/dL)	187.89 ± 2.83 (266)	71–357	196.35 ± 4.12 (114)	71–345	181.55 ± 3.81 (152)	76–357
HDL cholesterol (mg/dL)	41.87 ± 0.80 (266)	18–90	46.10 ± 1.22 (115)	18–85	38.65 ± 0.98 (151)	18–90
LDL cholesterol (mg/dL)	113.90 ± 2.29 (261)	24–254	117.21 ± 3.22 (115)	34–240	111.29 ± 3.21 (146)	24–254
Triglycerides (mg/dL)	176.58 ± 7.03 (273)	9–1215	167.87 ± 8.12 (119)	9–580	183.30 ± 10.77 (154)	9–1215

**Table 2:** Pathophysiological components and risk factors of the cardiometabolic syndrome in individuals of the study group. Number of cases is reported and percentages are given in parentheses.

	All	Males	Females
Body mass index > 30 (kg/m^2^)	132 (49%)	66 (44%)	66 (56%)
HDL cholesterol < 40 (mg/dl)	128 (48%)	94 (62%)	34 (30%)
Fasting glycemia > 125 mg/dl	36 (16%)	16 (13%)	20 (20%)
Type 2 diabetes	46 (17%)	23 (15%)	23 (19%)
Hypertension	147 (54%)	73 (47%)	74 (62%)
Hyperlipidemia	119 (44%)	67 (44%)	52 (44%)
Family history of hypertension	187 (69%)	99 (64%)	88 (74%)
Family history of type 2 diabetes	155 (57%)	83 (54%)	72 (61%)

**Table 3:** Genetic control of lipidomic signals mapped to the genome and proposed lipid assignments. Lipidome data, acquired with a Q Exactive Hybrid Quadrupole-Orbitrap mass spectrometer fitted with a Waters Acquity CSH C18 column, were tested for genetic association with genotyped SNPs in the study group (*n* = 273). Features were characterized by their retention time (RT) and their mass-to-charge ratio (*m*/*z*). Details of SNPs and statistical significance of lipidome features under monogenic control are reported. Full list of genetically mapped LC–MS lipidomic features and details and distinct SNP markers associated with lipid features under polygenic control are given in Supplementary Table 2. Candidate lipids proposed for lipidome features were identified through the LIPID MAPS Structure Database (https://www.lipidmaps.org, accessed on 4 June 2022). CAR, Acyl carnitine; DG, Diacylglycerol; FA, Fatty acyl; FOH, Fatty alcohol; LPA, Lipophosphatydicacid; LPC, Lysophosphatidylcholine; MG, Monoradylglycerol; NAE, *N*-acyl ethanolamine; PA, Phosphatidic acid; PC, Phosphatidylcholine; PE, Phosphatidylethanolamine; PS, Phosphatidylserine; ST, Sterol lipid; TG, Triacylgycerol; WE, Wax ester.

Positive-Ionization Mode					
*m*/*z*	RT	Genetic Control	Closest Marker	Closest Gene	Putative Lipid
204.123	37.098	Monogenic	rs6992234 (c8)	PSD3	CAR 2:0 (C9H17NO4)
277.216	67.495	Polygenic	-		FA 18:4 (C18H28O2), ST 18:1;O2 (C18H28O2), FA 18:3;O (C18H30O3)
279.232	66.953	Monogenic	rs7759479 (c6)	DST	FA 17:4 (C17H26O2)
295.227	67.515	Polygenic	-		FA 18:3;O (C18H30O3), FA 18:2;O2 (C18H32O4)
303.232	72.294	Polygenic	-		FA 20:5 (C20H30O2), ST 20:2;O2 (C20H30O2), FA 20:4;O (C20H32O3)
305.247	74.887	Polygenic	-		FA 20:4 (C20H32O2), ST 20:1;O2 (C20H32O2),FA 20:3;O (C20H34O3)
319.226	66.276	Polygenic	-		FA 20:5;O (C20H30O3), FA 20:4;O2 (C20H32O4)
343.224	71.225	Polygenic	-		FA 20:4;O (C20H32O3Na)
344.279	52.103	Monogenic	rs6928180 (c6)	GRIK2	CAR 12:0 (C19H37NO4), FA 19:2;O2 (C19H34O4), FOH 19:3;O3 (C19H34O4)
356.388	76.354	Polygenic	-		-
370.295	56.145	Monogenic	rs6928180 (c6)	GRIK2	CAR 14:1 (C21H39NO4), CAR 14:0;O (C21H41NO5), FA 21:3;O2 (C21H36O4)
377.266	110.856	Monogenic	rs1009439 (c6)	RCAN2	FA 21:2;O2 (C21H38O4Na), MG 18:2 (C21H38O4Na)
379.282	145.907	Monogenic	rs1009439 (c6)	RCAN2	FA 21:1;O2 (C21H40O4Na), MG 18:1 (C21H40O4Na), WE 21:1;O2 (C21H40O4Na)
398.326	67.497	Monogenic	rs6928180 (c6)	GRIK2	-
400.342	82.533	Monogenic	rs6928180 (c6)	GRIK2	CAR 16:0 (C23H45NO4), FA 23:2;O2 (C23H42O4)
426.357	88.672	Monogenic	rs6928180 (c6)	GRIK2	CAR 18:1 (C25H47NO4), CAR 18:0;O (C25H49NO5)
429.373	309.265	Polygenic	-		ST 29:2;O2 (C29H48O2), ST 29:1;O3 (C29H50O3)
431.352	314.575	Polygenic	-		ST 28:2;O3 (C28H46O3), ST 28:1;O4 (C28H48O4)
447.347	365.330	Polygenic	-		ST 28:2;O4 (C28H46O4), ST 28:1;O5 (C28H48O5)
448.391	309.387	Polygenic	-		-
469.365	309.438	Polygenic	-		ST 29:1;O3 (C29H50O3Na)
518.324	63.675	Polygenic	-		LPC 18:3 (C26H48NO7P), PC 18:1 (C26H50NO8P)
563.551	133.091	Polygenic	-		-
568.340	67.238	Monogenic	rs12997234 (c2)	DPP10	LPC 22:6 (C30H50NO7P)
590.321	67.252	Monogenic	rs12997234 (c2)	DPP10	LPC 22:6 (C30H50NO7PNa)
612.556	808.044	Monogenic	rs11855528 (c15)	CEMIP	DG 34:1 (C37H70O5), DG 35:2 (C37H70O5)
646.031	58.383	Polygenic	-		-
662.025	62.334	Polygenic	-		-
712.645	897.105	Monogenic	rs2002218 (c3)	IQSEC1	TG 40:0 (C43H82O6)
738.660	898.395	Polygenic	-		TG 42:1 (C45H84O6)
756.553	408.519	Polygenic	-		PC 34:3 (C42H78NO8P),PE 37:3 (C42H78NO8P), PS O-36:2 (C42H80NO9P), PA 39:4 (C42H75O8P)
758.560	408.446	Polygenic	-		-
758.569	457.168	Polygenic	-		PC 34:2 (C42H80NO8P), PC 37:2 (C42H80NO8P), PS O-36:1 (C42H82NO9P), PA 39:3 (C42H77O8P)
766.574	442.363	Monogenic	rs13362253 (c5)	MSX2	PC O-36:5 (C44H80NO7P), PC 36:3 (C44H82NO8P), PE 39:3 (C44H82NO8P)
780.553	373.605	Monogenic	rs2260930 (c20)	SEL1L2	PC 36:5 (C44H78NO8P), PE 39:5 (C44H78NO8P), PC 36:4;O (C44H80NO9P), PS O-38:4 (C44H80NO9P), PA 41:6 (C44H75O8P)
784.584	560.683	Polygenic	-		PC 36:3 (C44H82NO8P), PE 39:3 (C44H82NO8P), PA 41:4 (C44H79O8P)
792.707	921.958	Polygenic	-		TG 46:2 (C49H90O6)
864.764	887.193	Polygenic	-		-
876.728	841.945	Polygenic	-		-
886.749	911.605	Polygenic	-		-
888.764	928.842	Polygenic	-		-
890.771	929.103	Polygenic	-		-
894.754	922.854	Polygenic	-		TG 54:7 (C57H96O6)
912.764	912.510	Polygenic	-		-
914.779	929.523	Polygenic	-		-
922.785	939.142	Monogenic	rs2292329 (c16)	NECAB2	TG 56:7 (C59H100O6)
932.864	1004.391	Monogenic	rs11071737 (c15)	RAB8B	TG 56:2 (C59H110O6)
946.785	930.853	Polygenic	-		TG 58:9 (C61H100O6)
948.800	946.043	Polygenic	-		TG 58:8 (C61H102O6)
Negative-Ionization Mode					
187.006	36.489	Polygenic	-		-
271.228	113.649	Polygenic	-		FA 16:0;O (C16H32O3)
293.213	64.408	Polygenic	-		FA 18:3;O (C18H30O3)
295.228	64.394	Monogenic	rs7760515 (c6)	DST	FA 18:2;O (C18H32O3)
303.233	129.783	Polygenic	-		ST 20:1;O2 (C20H32O2)
311.223	64.059	Polygenic	-		FA 18:2;O2 (C18H32O4), FA 17:2 (C17H30O2), WE 17:2 (C17H30O2), WE 16:2 (C16H28O2), FA 16:2 (C16H28O2)
317.212	62.651	Monogenic	rs7193436 (c16)	MVD	FA 20:5;O (C20H30O3), ST 19:2;O (C19H28O)
319.228	70.158	Polygenic	-		FA 20:4;O (C20H32O3), ST 19:1;O (C19H30O)
321.243	71.306	Polygenic	-		FA 20:3;O (C20H34O3), ST 19:0;O (C19H32O)
327.233	118.705	Polygenic	-		FA 22:6 (C22H32O2)
343.228	65.947	Polygenic	-		FA 22:6;O (C22H32O3), ST 22:3;O3 (C22H32O3), ST 20:3;O (C20H28O)
345.244	68.352	Polygenic	-		ST 21:2;O (C21H32O), ST 20:2;O (C20H30O)
409.236	80.634	Polygenic	-		LPA 16:0 (C19H39O7P)
433.236	68.781	Polygenic	-		LPA 18:2 (C21H39O7P)
437.291	60.227	Polygenic	-		ST 24:1;O4 (C24H40O4),FA 23:4;O2 (C23H38O4),FOH 23:5;O3 (C23H38O4),MG 20:4 (C23H38O4),ST 23:1;O4 (C23H38O4)
446.377	287.415	Polygenic	-		NAE 24:0 (C26H53NO2), TG 55:5 (C58H102O6)
448.307	47.807	Polygenic	-		ST 24:1;O4;G (C26H43NO5)
457.236	66.170	Polygenic	-		ST 24:2;O6 (C24H38O6)
591.391	200.190	Polygenic	-		ST 27:2;O;Hex (C33H54O6)
605.406	223.252	Monogenic	rs1487842 (c11)	SYT9	ST 27:2;O;Hex (C33H54O6)
612.331	64.327	Monogenic	rs12997234 (c2)	DPP10	LPC 22:6 (C30H50NO7P),LPE 24:6 (C29H48NO7P)
804.567	435.379	Monogenic	rs2655474 (c9)	ELAVL2	PC O-36:3 (C44H84NO7P)
812.582	530.577	Polygenic	-		PC O-36:4 (C44H82NO7P), PC O-35:4 (C43H80NO7P), PE O-38:4 (C43H80NO7P)
828.577	487.561	Polygenic	-		-
828.577	514.160	Polygenic	-		-

**Table 4:** Significant associations between lipidomic features and clinical and biochemical phenotypes in the study group. Lipidomic features were independently acquired in negative- and positive-ionization modes in serum samples from a study group of 273 individuals. Linear regression was used to compute a P-value statistic for each metabolic feature, which was corrected for multiple testing using the Benjamini-Hochberg method to calculate adjusted *p*-values. Significant evidence of association was obtained for cardiometabolic disease (CMD), family history (FH) of hypertension, body-mass index (BMI) and total and HDL cholesterol. CMD was assessed by presence of at least three anomalies (diabetes, hypertension, BMI > 30kg/m^2^, HDL < 40mg/dl). Results from association analysis for all phenotypes that did not reach statistical significance following correction for multiple testing (nominal *p*-value < 0.05) are shown in Supplementary Table S3. Mass-to-charge ratio (*m*/*z*) and retention time (RT) are reported for each lipidome feature. Assignment of lipid candidates for lipidome features was performed using LIPID MAPS (https://www.lipidmaps.org, accessed 1 May 2022). CAR, Acyl carnitine; FA, Fatty acyl; CL, Cardiolipin; NAT, N-acyl amide; PE, Phosphatidylethanolamine; PG, Phosphatidylglycerol; ST, Sterol lipid.

	Ionization Mode	*m*/*z*	RT	P	Adjusted P	Correlation	R Squared	Adjusted R Squared	Putative Lipid
CMD	Negative	317.059	48.745	6.19 × 10^−9^	6.09 × 10^−6^	0.105	0.125	0.115	-
	Negative	319.056	48.759	7.97 × 10^−9^	6.09 × 10^−6^	0.061	0.123	0.113	-
	Negative	386.237	59.845	6.06 × 10^−8^	3.09 × 10^−5^	0.058	0.112	0.102	NAT 18:2 (C20H37NO4S)
	Negative	466.308	161.781	8.74 × 10^−7^	2.74 × 10^−4^	0.059	0.102	0.092	CAR 18:3 (C25H43NO4)
	Negative	465.305	162.010	1.02 × 10^−6^	2.74 × 10^−4^	0.053	0.103	0.093	ST 27:1;O;S (C27H46O4S)
	Negative	497.122	48.707	1.07 × 10^−6^	2.74 × 10^−4^	0.133	0.093	0.083	-
	Negative	231.021	48.730	7.22 × 10^−6^	0.002	0.015	0.080	0.070	FA 7:4;O4 (C7H6O6)
	Negative	233.018	48.759	8.94 × 10^−6^	0.002	0.150	0.079	0.068	-
	Negative	313.239	115.077	1.44 × 10^−5^	0.002	0.127	0.084	0.073	-
	Negative	463.344	138.712	9.16 × 10^−5^	0.014	0.016	0.057	0.046	ST 28:1;O5 (C28H48O5),ST 27:1;O3 (C27H46O3),ST 26:1;O3 (C26H44O3)
	Negative	551.359	180.907	2.40 × 10^−4^	0.033	0.140	0.071	0.061	-
	Negative	591.391	200.190	2.85 × 10^−4^	0.036	0.127	0.056	0.046	ST 27:2;O;He × (C33H54O6)
	Negative	592.394	200.009	3.79 × 10^−4^	0.043	0.124	0.055	0.045	PE 25:0 (C30H60NO8P)
	Negative	607.386	200.303	3.91 × 10^−4^	0.043	0.114	0.047	0.036	ST 27:1;O;GlcA (C33H54O7)
FH Hypertension	Negative	695.511	336.990	7.62 × 10^−6^	0.012	0.029	0.093	0.083	-
	Negative	938.536	440.693	3.61 × 10^−5^	0.028	0.104	0.068	0.058	-
BMI	Positive	774.543	527.985	1.80 × 10^−5^	0.027	0.182	0.091	0.081	-
	Positive	833.588	430.188	5.81 × 10^−5^	0.037	0.174	0.070	0.060	PG 40:4 (C46H83O10PLi)
	Positive	834.591	429.747	9.24 × 10^−5^	0.037	0.169	0.068	0.057	Hex 2Cer 32:1;O2 (C44H83NO13)
	Positive	832.584	429.512	9.85 × 10^−5^	0.037	0.161	0.064	0.053	PC 40:7 (C48H82NO8P), PS O-42:6 (C48H84NO9P)
Total Cholesterol	Positive	758.569	457.168	1.26 × 10^−6^	0.002	−0.012	0.085	0.075	-
	Positive	759.572	457.370	2.35 × 10^−6^	0.002	0.022	0.084	0.074	CL 76:2 (C85H162O17P2)
HDL Cholesterol	Negative	367.228	84.969	2.44 × 10^−5^	0.037	0.010	0.078	0.068	ST 24:5;O3 (C24H32O3)
	Positive	213.146	49.562	5.72 × 10^−6^	0.008	0.013	0.091	0.081	FA 13:4 (C13H18O2Li),WE 13:4 (C13H18O2Li)

## Data Availability

Data is contained within the article or supplementary material. The data presented in this study are available in Supplementary Tables.

## Supplementary Materials

The following supporting information can be downloaded at: https://www.mdpi.com/article/10.3390/metabo12070596/s1. Figure S1: 2-D Principal component analysis of mass spectrometry data in the cohort representing the scores of the first components; Table S1: Lipidome spectral features acquired in serum samples from the study population; Table S2: List of all SNPs showing evidence of statistically significant association with lipidome fearures; Table S3: Associations between lipidomic features and clinical and biochemical data.
