# Supplementary material for: Genetic Architecture of Untargeted Lipidomics in Cardiometabolic-Disease Patients Combines Strong Polygenic Control and Pleiotropy
Source: Metabolites. 2022 Jun 27;12(7):596. doi: 10.3390/metabo12070596 (PMC9322850; doi:10.3390/metabo12070596)
Supplement: Supplementary file 1 [file metabolites-12-00596-s001.zip › metabolites-1780948-Figure S1.pdf]

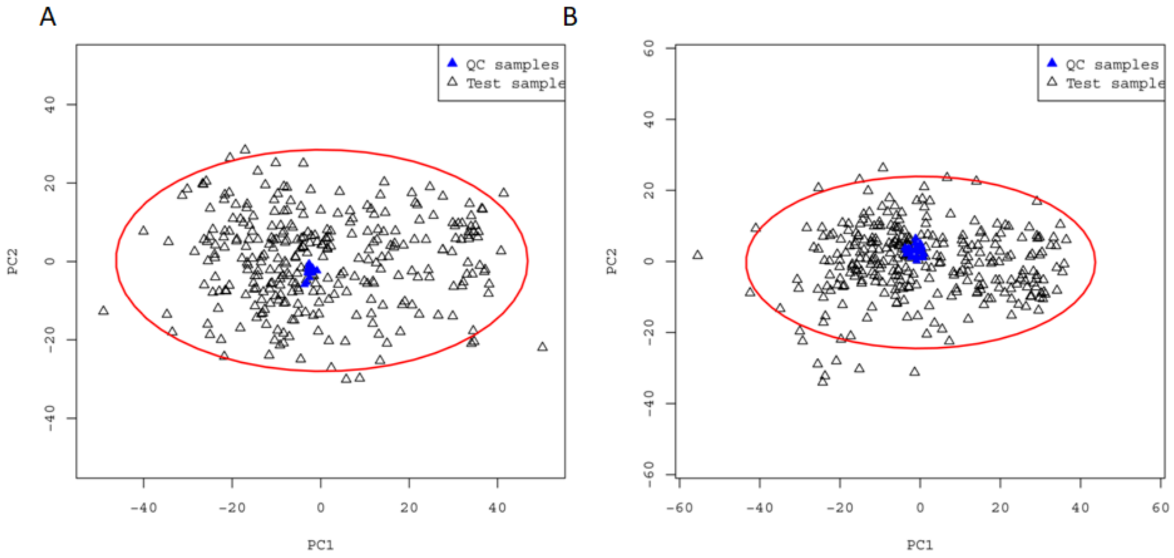

Figure S1. 2-D Principal component analysis of mass spectrometry data in the cohort representing the scores of the first components. Metabolome data from plasma samples of cohort processed with a CSH C18 column were analyze after filtering and normalization for the positive mode (A) and negative mode (B).
